# Assessing Plasma Levels of α-Synuclein and Neurofilament Light Chain by Different Blood Preparation Methods

**DOI:** 10.3389/fnagi.2021.759182

**Published:** 2021-11-08

**Authors:** Kuo-Hsuan Chang, Kou-Chen Liu, Chao-Sung Lai, Shieh-Yueh Yang, Chiung-Mei Chen

**Affiliations:** ^1^Department of Neurology, Chang Gung Memorial Hospital, Chang Gung University College of Medicine, Taoyuan, Taiwan; ^2^Department of Electronic Engineering, Artificial Intelligence and Green Technology Research Center, Chang Gung University, Taoyuan, Taiwan; ^3^Division of Pediatric Infectious Disease, Department of Pediatrics, Chang Gung Memorial Hospital, Taoyuan, Taiwan; ^4^Department of Nephrology, Chang Gung Memorial Hospital, Taoyuan, Taiwan; ^5^Department of Materials Engineering, Ming Chi University of Technology, New Taipei City, Taiwan; ^6^MagQu Co., Ltd., Taipei, Taiwan

**Keywords:** Parkinson’s disease, biomarker, α-synuclein, neurofilament light chain, ethylenediaminetetraacetic acid, centrifugation temperature

## Abstract

The potential biomarkers of Parkinson’s disease are α-synuclein and neurofilament light chain (NFL). However, inconsistent preanalytical preparation of plasma could lead to variations in levels of these biomarkers. Different types of potassium salts of EDTA and different centrifugation temperatures during plasma preparation may affect the results of α-synuclein and NFL measurements. In this study, we prepared plasma from eight patients with Parkinson’s disease (PD) and seven healthy controls (HCs) by using di- and tri-potassium (K_2_- and K_3_-) EDTA tubes and recruited a separated cohort with 42 PD patients and 40 HCs for plasma samples prepared from whole blood by centrifugation at room temperature and 4°C, respectively, in K_2_-EDTA tubes. The plasma levels of α-synuclein and NFL in K_2_- and K_3_-EDTA were similar. However, the levels of α-synuclein in the plasma prepared at 4°C (101.57 ± 43.43 fg/ml) were significantly lower compared with those at room temperature (181.23 ± 196.31 fg/ml, *P* < 0.001). Room temperature preparation demonstrated elevated plasma levels of α-synuclein in PD patients (256.6 ± 50.2 fg/ml) compared with the HCs (102.1 ± 0.66 fg/ml, *P* < 0.001), whereas this increase in PD was not present by preparation at 4°C. Both plasma preparations at room temperature and 4°C demonstrated consistent results of NFL, which are increased in PD patients compared with HCs. Our findings confirmed that K_2_- and K_3_-EDTA tubes were interchangeable for analyzing plasma levels of α-synuclein and NFL. Centrifugation at 4°C during plasma preparation generates considerable reduction and variation of α-synuclein level that might hinder the detection of α-synuclein level changes in PD.

## Introduction

The development of biomarker research has driven a new avenue toward the diagnosis and prediction of the progression of Parkinson’s disease (PD). PD is clinically characterized by motor dysfunction including bradykinesia, rigidity, tremor, postural instability, and freezing of gait, as well as pathologically by the progressive loss of dopaminergic neurons in substantia nigra (Lang and Lozano, 1998). To assess disease severity in PD, different rating tools, such as the unified PD rating scale (UPDRS) or Hoehn and Yahr stage (Hoehn and Yahr, 1967; Movement Disorder Society Task Force on Rating Scales for Parkinson’s Disease, 2003), are widely used in clinical practice. However, these scales demonstrate significant inter- and intra-rater variability (Shulman et al., 2010). Their non-linear properties further limit the application of these scales to precisely assess the progression of neurodegeneration. Therefore, discovery and validation of PD-specific molecular biomarkers, particularly in body fluids such as blood and cerebrospinal fluid (CSF), are important to provide objective and linear tools for diagnosing the disease at prodromal or early stages, as well as monitoring disease progression and therapeutic responses.

Among biomarker candidates for neurodegenerative diseases, α-synuclein, and neurofilament light chain (NFL) are the two most studied molecules in PD. The α-synuclein is a major constituent of Lewy bodies, the pathological intracytoplasmic inclusions in degenerative dopaminergic neurons of PD patients (Spillantini et al., 1997). As highly expressed in large-caliber myelinated axons, NFL has been suggested to be involved in axonal injury and degeneration (Petzold, 2005). Given that abnormal accumulation of α-synuclein and NFL in body fluids may reflect the abnormalities in the brains of PD patients, these proteins have gained attention as potential surrogate biomarkers for PD (Simonsen et al., 2016). Compared with CSF, plasma is relatively easy to access and preferable for biomarker analysis. However, the determinations of α-synuclein and NFL in plasma yield conflicting results. Plasma α-synuclein levels in PD patients could be increased (Lee et al., 2006; Ding et al., 2017; Lin et al., 2017; Chang et al., 2019; Ng et al., 2019), decreased (Li et al., 2007), or unchanged (Mata et al., 2010; Park et al., 2011; Foulds et al., 2013). NFL levels in the plasma of PD patients could be elevated (Lin et al., 2019), or modest increases in PD only in one of the cohorts (Hansson et al., 2017). The inconsistency of preanalytical sample preparations may affect the measurement results of α-synuclein and NFL in plasma.

Components in the blood collection tubes, such as anticoagulants, gels, and clot activators, have been recognized as significant sources, resulting in variability in the quantification of molecules in the blood (Bowen and Remaley, 2014). Plasma is usually prepared from whole blood in an EDTA-containing tube. Di- and tri-potassium (K_2_ and K_3_) salts of EDTA are the standard anticoagulants used for plasma preparations. It has been recognized that the type of EDTA salts may affect the accuracy of cell counting and sizing (Goossens et al., 1991). K_2_-EDTA was suggested as the anticoagulant of choice for routine hematology testing in hospitals (England et al., 1993). However, K_3_-EDTA tubes were used in a few biomarker studies for PD (Lin et al., 2017; Chang et al., 2019). On the other hand, centrifugation condition for quick separation of cells from the plasma is also crucial. It is recommended that plasma is ideally centrifuged at room temperature (RT) for hemostasis assays (Adcock Funk et al., 2012). Some laboratories prefer to process blood samples at refrigerated temperatures at 4°C (Mussbacher et al., 2017). In this study, we investigated whether K_2_-EDTA tubes introduced any significant bias in α-synuclein and NFL measurements as compared with K_3_-EDTA tubes. We further examined the effect of centrifugation temperatures during plasma preparation on the measurements of α-synuclein and NFL.

## Materials and Methods

### Ethics Approval and Consent to Participate

This study was approved by the Institutional Review Boards of the Chang Gung Memorial Hospital (ethical license No: 201801049A3 and 201801051A3).

### Patient Recruitment

This was a cross-sectional study and patients were recruited from July 1, 2018, to December 31, 2020, in Chang Gung Memorial Hospital-Linkou Medical Center in Taiwan. Patients were diagnosed as PD according to the UK Brain Bank criteria for PD (Hughes et al., 1992). UPDRS (Movement Disorder Society Task Force on Rating Scales for Parkinson’s Disease, 2003), Hoehn and Yahr stage (Hoehn and Yahr, 1967), levodopa equivalent daily dose (LEDD) (Tomlinson et al., 2010), mini-mental state examination (Tombaugh and McIntyre, 1992), and demographic information was recorded for each patient. PD patients with Hoehn and Yahr stages 1–2 were defined as at the early stage, while those with Hoehn and Yahr stages higher than 2 were classified as at the advanced stage. Sex/age-matched healthy control (HC) subjects were randomly recruited from neurology outpatient clinics. The HC subjects visiting neurology outpatient clinics were diagnosed with insomnia, tension headache, myalgia, or low back pain. All HC subjects had no systemic infection, chronic renal failure, cardiac or liver dysfunction, malignancies, autoimmune diseases, PD, stroke, or any neurodegenerative diseases. Diagnoses were determined by two experienced neurologists in movement disorders (KH Chang and CM Chen) who were blinded to both α-synuclein and NFL levels in plasma. This was a prospective study. For the first part experiment of K_2_- and K_3_-EDTA tube comparison, the first eight PD patients were recruited and the seven age- and gender-matched HC were selected accordingly. Subsequently, all the 42 PD patients and the 40 age- and gender-matched HC including the subjects from the first part were included for the second part of the study in assessing sample centrifugation under RT or 4°C.

### Sample Collection and Preparation

Ten-milliliter K_2_- (BD vacutainer K2, 367525, BD, Franklin Lakes, NJ) or K_3_-EDTA tubes (455036, Greiner Bio-One GmbH, Kremsmunster, Austria) were used for the blood draw. After blood collection, the blood collection tube was gently inverted 10 times immediately. Blood collection tubes were immediately centrifuged at 1,500–2,500 g for 15 min within 1 h. In this study, each participant was requested to donate two tubes of blood. One was centrifuged at RT, the other was centrifuged at 4°C. A swing-out (basket) rotor was used for centrifugation. By using a disposable 1 ml micropipette tip, every 1 ml of plasma (supernatant) was transferred to a fresh 1.5 ml Eppendorf tube. All plasma samples were frozen at –80°C before measurements.

### Measurement of α-Synuclein and Neurofilament Light Chain Levels in Plasma

We used an immunomagnetic reduction (IMR) assay to measure the plasma levels of total α-synuclein and NFL (Yang et al., 2016). The frozen human plasma sample was moved from –80°C to wet ice, and then to RT for 20 min. Afterward, 40 μl of plasma was mixed with 80 μl of reagent (MF-ASCd with MagQu, New Taipei City, Taiwan) for total α-synuclein assay, while 60 μl of plasma was mixed with 60 μl of reagent (MF-NFLd with MagQu, New Taipei City, Taiwan) for NFL. For each batch of measurements, calibrators (CA-DEX-0060, CA-DEX-0080, MagQu, New Taipei City, Taiwan) and control solutions (CL-ASC-000T, MagQu, New Taipei City, Taiwan) were used. The IMR analyzer (XacPro-S361, MagQu, New Taipei City, Taiwan) was utilized. For each sample, duplicated measurements were performed for assaying total α-synuclein and NFL. The averaged concentration of the duplicated measurements was reported. Plasma total α-synuclein and NFL levels were determined by technicians who were blinded to the clinical diagnosis. The limit of detection (LoD) and analytical range (total α-synuclein: 0.0014–1,020 pg/ml; NFL: 0.001–1,000 pg/ml) were determined according to Clinical and Laboratory Standards Institute (CLSI)/National Committee for Clinical Laboratory Standards (NCCLS) document, Evaluation of Detection Capability for Clinical Laboratory Measurement Procedures (EP17-A2) and Validation of analytical procedure [ICH Q2(R1)], respectively. For intra- (repeatability) and inter-assay (reproducibility), the precision testing was determined according to CLSI/NCCLs document Evaluation of Precision of Quantitative Measurement Procedures (EP5-A3). Two samples with different total α-synuclein and NFL concentrations were measured. The measurements of each concentration had been conducted a total of 80 times. Inter- (repeatability) and intra-assay (reproducibility) involved assaying two samples on 20 different working days, with two runs per day, and two replicates per run, i.e., a 20 × 2 × 2 design. The coefficient variances of inter- (repeatability) and intra-assay (reproducibility) of the total α-synuclein and NFL IMR assay are shown in Supplementary Table 1.

### Measurement of Hemoglobin Level in Plasma

Hemoglobin was estimated by the cyanmethemoglobin methods of Drabkin (Sigma). Readings were taken at 540 nm in a spectrophotometer.

### Statistical Analysis

The Statistical Program for Social Sciences [SPSS version 23 (IBM, Armonk, NY) and GraphPad Prism 8 (GraphPad, La Jolla, CA) were used to analyze all the statistics]. Data distribution normality was examined by D’Agostino-Pearson omnibus test. Mann-Whitney *U*-test, or Kruskal-Wallis test followed by Dunn *post hoc* test, was applied to compare the differences of two non-categorical variables whose distribution is not Gaussian. For the variables with Gaussian distribution, differences between two groups were examined by Student’s *t-*test or analysis of covariance (ANCOVA), as appropriate. Pearson’s correlation analysis was performed to analyze the linear correlation between two variables. Bland-Altman plotting was performed to assess the comparability of results obtained by different methods. Sex distribution was analyzed by χ^2^-test. To examine the diagnostic accuracy of plasma α-synuclein and NFL levels, the receiver operating characteristic (ROC) curve was used to determine the area under the ROC curve (AUC) of variables, and the values of sensitivity and specificity. Each set of data was expressed as median ± *SD*. All *P*-values were two-tailed, and a *P* < 0.05 was considered significant.

## Results

**FIGURE 1:**
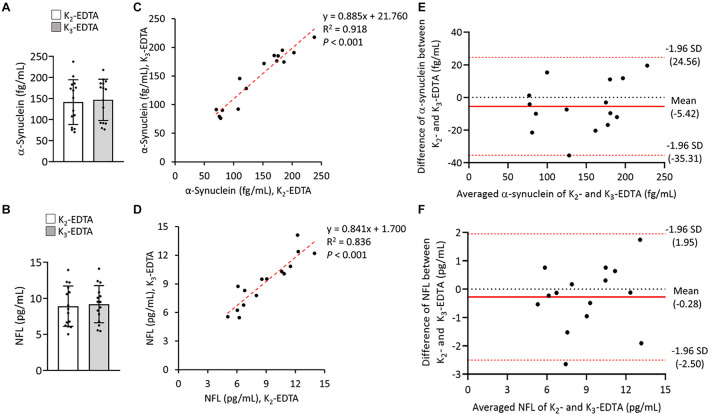
Levels of α-synuclein and neurofilament light chain (NFL) in the plasma were prepared by using different EDTA tubes. **(A)** Plasma levels of α-synuclein in K_2_- (*N* = 15) and K_3_- (*N* = 15) EDTA tubes. **(B)** Plasma levels of NFL in K_2_- and K_3_-EDTA tubes. **(C)** Correlations between α-synuclein levels in K_2_- and K_3_-EDTA tubes. **(D)** Correlations between NFL levels in K_2_- and K_3_-EDTA tubes. **(E)** Bland-Altman plot of the comparability of α-synuclein levels in K2- and K3-EDTA tubes. The red dotted lines represent limits of agreement (mean difference ± 1.96 *SD*). **(F)** Bland-Altman plot of the comparability of NFL levels in K2- and K3-EDTA tubes. *R*^2^, Pearson’s correlation coefficient. Comparisons by Mann-Whitney *U*-test.

**FIGURE 2:**
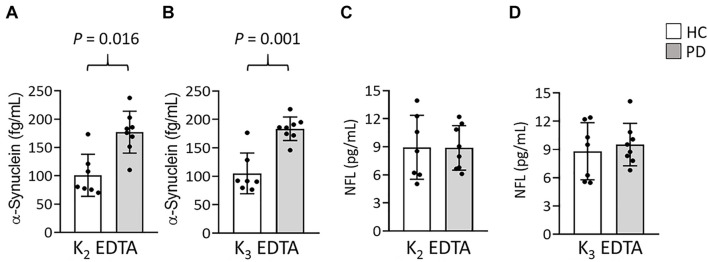
Levels of α-synuclein and NFL in the plasma of patients with Parkinson’s disease (PD) and healthy controls (HCs). **(A,B)** Plasma levels of α-synuclein in patients with PD (*n* = 8) and healthy controls (HCs, *n* = 7) in **(A)** K_2_- and **(B)** K_3_-EDTA tubes. **(C,D)** Plasma levels of NFL in patients with PD and HCs in **(C)** K_2_- and **(D)** K_3_-EDTA tubes. Error bars represent *SD*. Comparisons by Mann-Whitney *U*-test.

To compare the relative influences of different potassium salts of EDTA on the measurement of α-synuclein and NFL levels in plasma by IMR we collected blood by using K_2_ and K_3_-EDTA tubes at RT from 8 patients with PD and 7 HCs. The plasma levels of α-synuclein (K_2_: 141.5 ± 53.16 fg/ml, K_3_: 146.92 ± 49.06 fg/ml, *P* = 0.595, Figure 1A) and NFL (K_2_: 8.92 ± 2.8 pg/ml, K_3_: 9.19 ± 2.58 pg/ml, *P* = 0.744, Figure 1B) in K_2_- and K_3_-EDTA were similar. The effective size to detect the difference between the levels of these molecules in K_2_- and K_3_-EDTA tubes was 0.1 and with a power of 0.9, and a sample size of 1,043 was needed to achieve a significant difference. Significant correlations were also demonstrated between these levels in K_2_ and K_3_-EDTA (α-synuclein: *R*^2^ = 0.918, *P* < 0.001, Figure 1C; NFL: *R*^2^ = 0.836, *P* < 0.001, Figure 1D). Bland-Altman plotting also confirmed that the differences in the tested molecule levels between K_2_- and K_3_-EDTA collection were small (α-synuclein: –5.42 ± 15.3 fg/ml, Figure 1E; NFL: –0.28 ± 1.14 pg/ml, Figure 1F). Compared with HCs, a significantly higher levels of α-synuclein in PD patients were detected by using either K_2_- (PD: 177.1 ± 37.04 fg/ml, HC: 100.90 ± 37.16 fg/ml, *P* = 0.016, Figure 2A) or K_3_-EDTA (PD: 183.05 ± 20.79 fg/ml, HC: 105.10 ± 35.78 fg/ml, *P* = 0.001, Figure 2B) collection, although the number of cases was small. Plasma levels of NFL in PD and HCs were similar by using either K_2_- (PD: 8.89 ± 2.38 pg/ml, HC: 8.95 ± 3.42 pg/ml, *P* = 0.961, Figure 2C) or K_3_-EDTA collection (PD: 9.53 ± 2.26 pg/ml, HC: 8.81 ± 3.03 pg/ml, *P* = 0.61, Figure 2D). These results showed no remarkable differences in plasma levels of α-synuclein and NFL between K_2_- and K_3_-EDTA preparations.

**FIGURE 3:**
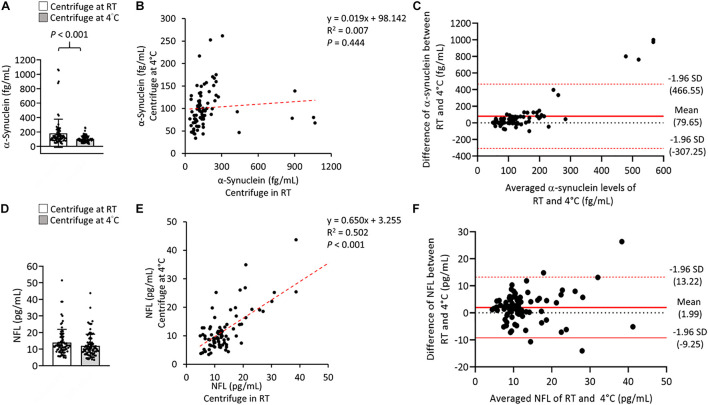
Levels of α-synuclein and NFL in the plasma were centrifuged at different temperatures. **(A)** Plasma levels and α-synuclein in K_2_-EDTA tubes were centrifuged at room temperature (RT) or 4°C. **(B)** Correlations between α-synuclein levels in K_2_-EDTA tubes centrifuged at RT or 4°C. **(C)** Bland-Altman plot of the comparability of α-synuclein levels in K_2_-EDTA tubes centrifuged at RT or 4°C. The red dotted lines represent limits of agreement (mean difference ± 1.96 *SD*). **(D)** Plasma levels and of NFL in K_2_-EDTA tubes centrifuged at RT or 4°C. **(E)** Correlations between NFL levels in K_2_-EDTA tubes centrifuged at RT or 4°C. **(F)** Bland-Altman plot of the comparability of α-synuclein levels in K_2_-EDTA tubes centrifuged at RT or 4°C. *R*^2^, Pearson’s correlation coefficient. Comparisons by Student’s *t-*test.

**FIGURE 4:**
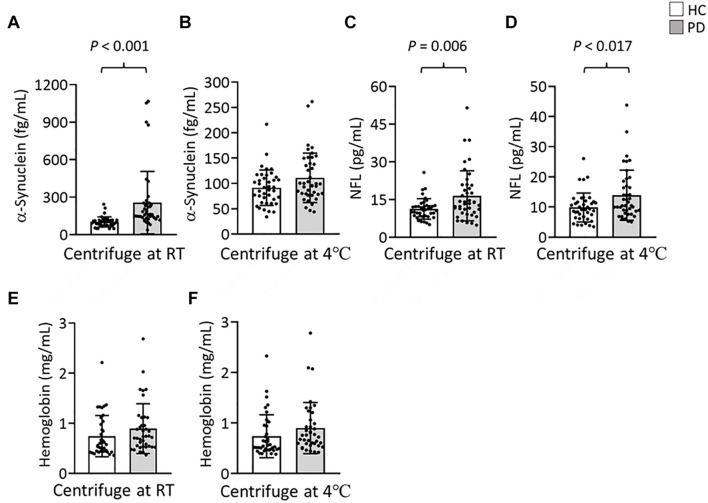
Levels of α-synuclein and NFL in the plasma of patients with PD and HCs. **(A,B)** Plasma levels of α-synuclein in patients with PD (*n* = 42) and HCs (*n* = 40) in K_2_-EDTA tubes centrifuged at **(A)** RT or **(B)** 4°C. **(C,D)** Plasma levels of NFL in patients with PD and HCs in K_2_-EDTA tubes centrifuged at **(C)** RT or **(D)** 4°C. **(E,F)** Plasma levels of hemoglobin in patients with PD and HCs in K_2_-EDTA tubes centrifuged at **(E)** RT or **(F)** 4°C. Error bars represent *SD*. Comparisons by Student’s *t-*test.

**FIGURE 5:**
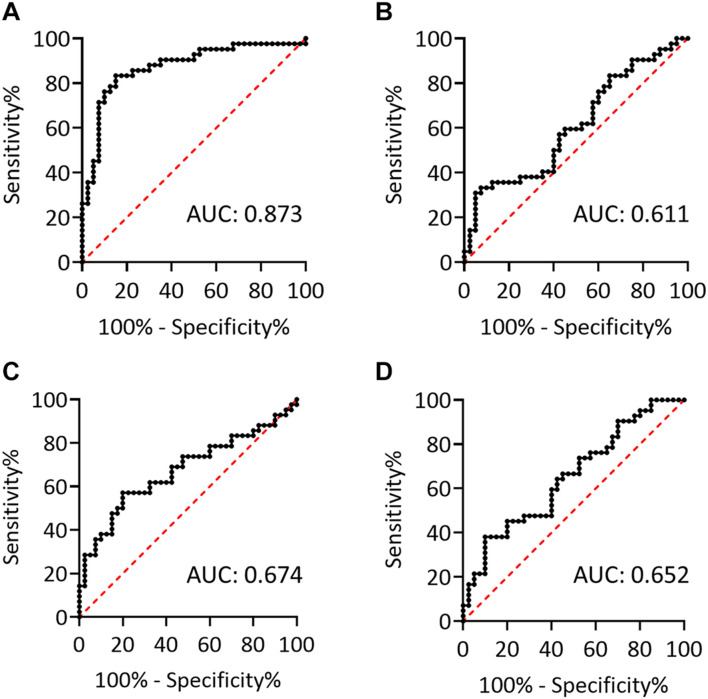
The potentials to discriminate PD and HCs. **(A,B)** Receiver operating characteristic (ROC) curves for plasma levels of α-synuclein in K_2_-EDTA tubes centrifuged at **(A)** RT or **(B)** 4°C to discriminate patients with PD from HCs. **(C,D)** ROC curves for plasma levels of NFL in K_2_-EDTA tubes centrifuged at **(C)** RT or **(D)** 4°C to discriminate patients with PD from HCs. AUC, area under the ROC curve.

To further evaluate the effect of centrifugation temperature on plasma levels of α-synuclein and NFL, we prepared plasma from 42 patients with PD and 40 HCs (Table 1) by using centrifuging blood samples in K_2_-EDTA at RT or refrigerated temperature. Under RT centrifugation, the levels of α-synuclein (*n* = 82, 181.23 ± 196.31 fg/ml) were significantly higher compared with refrigerated centrifugation (*n* = 82, 101.57 ± 43.43 fg/ml, *P* < 0.001, Figure 3A), and α-synuclein levels in plasma prepared from the two temperature conditions of centrifugation did not demonstrate a significant correlation (*R*^2^ = 0.007, *P* = 0.444, Figure 3B). Bland-Altman plotting also demonstrated a huge difference in α-synuclein levels between these two conditions (79.65 ± 197.4 fg/ml, Figure 3C). On the other hand, NFL levels in plasma prepared from RT and refrigerated centrifugations were similar (RT: 13.92 ± 8.05 pg/ml, 4°C: 11.94 ± 7.1 pg/ml, *P* = 0.1, Figure 3D). NFL levels in plasma under RT centrifugation were highly correlated with those under refrigerated centrifugation (*R*^2^ = 0.836, *P* < 0.001, Figure 3E). Bland-Altman plotting also confirmed that the differences between these two groups were small (1.99 ± 5.73 pg/ml, Figure 3F). Under RT centrifugation, the levels of α-synuclein in PD patients (256.6 ± 50.2 fg/ml) were significantly higher compared with HCs (102.1 ± 0.66 fg/ml, *P* < 0.001, Figure 4A). The sensitivity analysis confirmed the statistical significance following removing the outliers in PD group (PD: 181.06 ± 102.05 pg/ml, *P* < 0.001). However, this difference was not recapitulated with refrigerated centrifugation (PD: 111.1 ± 48.69 fg/ml; HC: 91.59 ± 35 fg/ml, *P* = 0.085, Figure 4B). Under both RT and refrigerated centrifugations, the levels of NFL in PD patients (RT: 16.47 ± 9.96 pg/ml; 4°C: 13.92 ± 8.31 pg/ml) were significantly higher compared with HCs (RT: 11.24 ± 4.06 pg/ml, *P* = 0.006, Figure 4C; 4°C: 9.84 ± 4.81 pg/ml, *P* = 0.017, Figure 4D). To further understand whether hemolysis could affect the levels of α-synuclein in plasma, we measured hemoglobin levels in our samples. The results showed hemoglobin levels in PD (RT:0.89 ± 0.5 mg/ml; 4°C:0.90 ± 0.56 mg/ml) and HCs (RT:0.74 ± 0.41 mg/ml, *P* = 0.15; 4°C:0.74 ± 0.43 mg/ml, *P* = 0.13) were similar in both temperatures of centrifugations (Figures 4E,F). The levels of α-synuclein between PD patients and HC under RT centrifugation remained significantly different after ANCOVA adjustment for hemoglobin levels (*P* < 0.001). AUC showed good ability for α-synuclein under RT centrifugation (AUC = 0.873, 95% CI: 0.793–0.953, Figure 5A) to distinguish PD patients from HCs, whereas α-synuclein levels under refrigerated centrifugation (AUC = 0.611, 95% CI: 0.488–0.733, Figure 5B), and NFL levels under RT (AUC = 0.674, 95% CI: 0.557–0.792, Figure 5C) and refrigerated centrifugation (AUC = 0.652, 95% CI: 0.535–0.77, Figure 5D) did not demonstrated adequate AUCs to separate PD from HCs.

**TABLE 1:** Clinical characteristics of the patients with Parkinson’s disease (PD) and healthy controls (HC).

	**HC (*n* = 40)**	**PD**		
		**Early (*n* = 24)**	**Advanced (*n* = 18)**	**Total (*n* = 42)**
Sex (female/male)	19/21	9/15	8/10	17/25
Age (years)	65.85 ± 7.52	61.58 ± 9.57	71.61 ± 9.15	65.88 ± 10.55
Duration (years)		5.78 ± 4.94	13.06 ± 6.58	8.88 ± 6.69
Hoehn and Yahr stage		1.54 ± 0.51	3.22 ± 0.43	2.17 ± 0.93
LEDD (mg)		549.73 ± 548.88	1422.69 ± 662.50	923.86 ± 736.32
UPDRS-total		23.38 ± 6.53	69.29 ± 21.71	42.37 ± 27.15
UPDRS-part III		13.92 ± 5.40	40.36 ± 12.54	24.88 ± 15.93
MMSE	29.62 ± 0.72	28.33 ± 2.76	22.59 ± 6.48	25.95 ± 5.42
CDR	0.13 ± 0.22	0.27 ± 0.25	0.59 ± 0.40	0.41 ± 0.36

**FIGURE 6:**
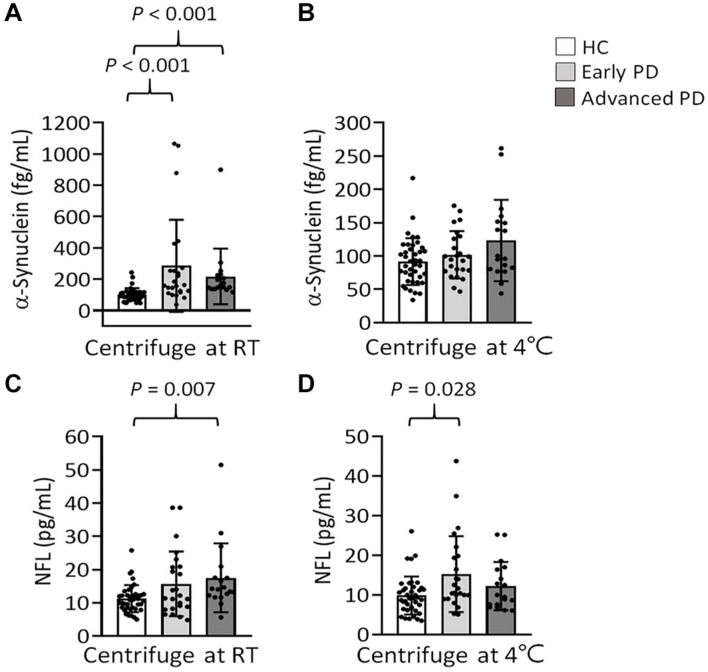
Levels of α-synuclein and NFL in the plasma of patients with PD at early or advanced stages, and HCs. **(A,B)** Plasma levels of α-synuclein in patients with early (*n* = 24) and advanced PD (*n* = 18), and HCs (*n* = 40) in K_2_-EDTA tubes centrifuged at **(A)** RT or **(B)** 4°C. **(C,D)** Plasma levels of NFL in patients with early and advanced PD, and HCs in K_2_-EDTA tubes centrifuged at **(C)** RT or **(D)** 4°C. Error bars represent *SD*. Comparisons by Kruskal-Wallis test followed by Dunn *post hoc* test.

We further stratified our patients into early and advanced PD according to their disease stages. Under RT centrifugation, the levels of α-synuclein among these groups were significant after ANCOVA adjustment for hemoglobin levels (*P* = 0.002). PD patients at early (285.64 ± 293.81 fg/ml, *P* < 0.001) and advanced PD (217.95 ± 177.41 fg/ml, *P* < 0.001) demonstrated higher levels of α-synuclein compared with HCs (102.05 ± 40.66 fg/ml, Figure 6A). These differences cannot be recapitulated under refrigerated centrifugation (early PD: 101.87 ± 35.63 fg/ml; advanced PD: 123.37 ± 61; HC: 91.59 ± 35.01, *P* = 0.157, Figure 6B). Under RT centrifugation, the levels of NFL (17.52 ± 10.37 pg/ml) in advanced PD were significantly higher compared with HCs (11.24 ± 4.06 pg/ml, *P* = 0.007, Figure 6C), but not significantly different between early PD (15.69 ± 9.74 pg/ml) and HCs (11.24 ± 4.06 pg/ml, *P* = 0.082). Under refrigerated centrifugation, early PD demonstrated significantly higher levels of NFL (15.23 ± 9.59 pg/ml) compared with HCs (9.84 ± 4.81 pg/ml, *P* = 0.028, Figure 6D), but not significantly different between advanced PD (12.22 ± 6.1 pg/ml) and HCs (9.84 ± 4.81 pg/ml, *P* = 0.669). Levels of α-synuclein and NFL in plasma under different centrifugation temperatures did not demonstrate correlations with the scores of UPDRS, Hoehn and Yahr stages or LEDDs (data not shown).

## Discussion

The α-synuclein and NFL levels in plasma have been widely tested to know if they can serve as potential biomarkers to indicate PD and disease progression, whereas the results are not satisfactorily consistent (Lee et al., 2006; Ding et al., 2017; Lin et al., 2017; Chang et al., 2019; Ng et al., 2019). It is noted that different assay methods including IMR, single-molecule array, and enzyme-linked immunosorbent assay may explain the different results between studies but using the same assay platform may also generate differential data caused by different preanalytical conditions. These include blood collection tubes, centrifuge, and storage temperatures. In order to identify useful markers to indicate disease onset, severity, and progression or to test the efficacy of potential treatments for future clinical trials, an optimized and standardized blood sample preparation are crucial to minimizing data variations from different centers. Therefore, the sample preparation methods should be taken into account for the establishment of reliable biochemical assessment of biomarkers for neurodegenerative diseases including PD. In this study, we evaluated the levels of α-synuclein and NFL in plasma, which were collected in K_2_- or K_3_-EDTA tubes and centrifuged at RT or refrigerated temperature. The results showed a linear relationship for the levels of α-synuclein and NFL between K_2_- or K_3_-EDTA tubes. The levels of α-synuclein in the plasma collected by both types of tubes were consistently elevated in PD patients compared with HCs. However, the temperature of centrifugation significantly affects the levels of α-synuclein. The levels of α-synuclein in the plasma prepared by RT centrifugation were not significantly correlated with those in the plasma prepared by refrigerated centrifugation. Similar to those reported in the literature (Lee et al., 2006; Ding et al., 2017; Lin et al., 2017; Chang et al., 2019; Ng et al., 2019), elevated levels of α-synuclein in PD patients were observed in plasma prepared by RT centrifugation, whereas such a change for PD was not present in plasma prepared by refrigerated centrifugation. The levels of NFL in plasma prepared by RT centrifugation demonstrated a linear relationship with those in plasma prepared by refrigerated centrifugation. Both conditions consistently demonstrated higher levels of NFL in PD patients compared with HCs. Our results suggested that the K_2_-EDTA tube was equivalent to the K_3_-EDTA tube in collecting blood to analyze plasma levels of α-synuclein and NFL. Centrifugation temperatures during plasma preparation generated a considerable variation of α-synuclein level that might hinder the identification of α-synuclein level changes in PD. Therefore, the choice of temperature for plasma preparation is crucial for assessing biomarkers in PD.

Plasma is usually prepared from blood in EDTA-anticoagulated tubes. EDTA comes in blood tubes as K_2_- and K_3_-EDTA salts. K_2_-EDTA is dispensed as powder, so it causes no variation in the volume/dilution of the collected sample. K_3_-EDTA is placed inside the tube in liquid form, which causes a slight dilution of the sample, possibly generates an osmotic effect on blood cells, and increases the variations in hemogram studies (Goossens et al., 1991). Prolonged storage of blood in K_3_-EDTA may reduce the size of red blood cells (RBCs) (Goossens et al., 1991). There is no significant difference in plasma glycohemoglobin levels between blood collections using K_2_- and K_3_-EDTA tubes (Vrtaric et al., 2016). In respect of biomarkers for Alzheimer’s disease, there was no marked difference in the levels of amyloid β and tau between K_2_- and K_3_-EDTA tubes (Rozga et al., 2019). Our results provide evidence for the small discrepancy between K_2_- and K_3_-EDTA tubes in measuring plasma levels of α-synuclein and NFL. The differences in α-synuclein between PD and HCs are also consistently found in K_2_- and K_3_-EDTA tubes, suggesting that both tubes can be used interchangeably for clinical assessments of biomarkers for PD.

Centrifugation is a necessary and key step in blood processing. Although it has been recommended that blood samples should be centrifuged at RT for homeostasis assays (Adcock Funk et al., 2012), there is no evidence that centrifugation temperatures might significantly influence concentrations of tested molecules in plasma. On routine coagulation tests, centrifugation of blood at refrigerated temperature is not likely to generate significant analytical or clinical biases on activated partial thromboplastin time, fibrinogen, and D-dimer (Lippi et al., 2006). However, our results showed refrigerated centrifugation significantly reduced the level of α-synuclein in plasma, as well as undermined the differences between PD and HCs. α-Synuclein is an amyloidogenic protein, which is prone to aggregate to form specific cross-β amyloid fibrils (Araki et al., 2019). The fibrillation formation and solubility of α-synuclein may be affected by temperatures. The inhibition of α-synuclein fibril formation appears after 60°C (Ariesandi et al., 2013), while the low temperature enhances the α-synuclein denaturation (Ikenoue et al., 2014). This cold denaturation is not seen in other amyloidogenic peptides, such as β2−microglobulin, amyloid β, and insulin (Ikenoue et al., 2014). Previous studies also showed that centrifugation temperature did not affect the levels of amyloid β in plasma (Verberk et al., 2020). Consistent with the study of Verberk, we found the level of NFL is not significantly biased by centrifugation temperatures (Verberk et al., 2020).

Lines of study have demonstrated elevation of α-synuclein in plasma of PD patients, using different assessment tools including enzyme-linked immunosorbent assay (Lee et al., 2006; Ding et al., 2017), single-molecule array (Ng et al., 2019), or IMR (Lin et al., 2017; Chang et al., 2019). Our results recapitulated this important finding by centrifuging blood at RT and demonstrated a good potential of α-synuclein in plasma to discriminate PD and HCs. However, a few studies report negative findings showing either decreased or unchanged α-synuclein levels (Mata et al., 2010; Park et al., 2011; Foulds et al., 2013). Given that 99% of α-synuclein in the blood is stored in RBCs (Barbour et al., 2008), potential hemolysis could confound the results. Blood sample preparations can be another important factor contributing to this inconsistency. This point is addressed by our results, which showed that refrigerated centrifugation reduced the level of α-synuclein, lessen the difference of α-synuclein levels between PD and HCs, and critically lowered the diagnostic potential of α-synuclein in PD. The potential mechanism underlying increased plasma levels of α-synuclein in PD remains unclear. Neurons that resided at the enteric plexus may harbor α-synuclein in PD patients since the prodromal stage (Stokholm et al., 2016). α-Synuclein released by peripheral neurons into the bloodstream may be transported to the brain and gradually seeds to form aggregations in central neurons. Therefore, assessment of α-synuclein in blood, with appropriate sample preparations and quantitative technologies, can offer a useful biomarker for the early diagnosis of PD. Further prospective studies, focusing on the patients at prodromal or early stages, may be needed to confirm the role of α-synuclein in the early diagnosis of PD.

Plasma NFL levels are elevated in several neurodegenerative diseases, including amyotrophic lateral sclerosis (Lu et al., 2015), multiple sclerosis (Piehl et al., 2018), Alzheimer’s disease (Weston et al., 2017), and atypical parkinsonian syndromes (Hansson et al., 2017). However, the results of blood NFL levels were not consistent. The study of Hansson et al. (2017) reported plasma NFL levels in patients with PD were modestly elevated in the London cohort but not in the Lund cohort. Similar to our results, the work of Lin et al. (2019) reported higher blood NFL levels in patients with PD, particularly at the advanced stage. It is of interest that plasma in both RT and refrigerated centrifugations demonstrated similar levels of NFL. It has been shown that blood levels of NFL may not be affected by repeated freeze-thaw cycles, delayed processing, and long-term storage at different temperatures, suggesting the NFL is stable under the common sample preparations (Barro et al., 2020). The study of Lin et al. (2019) also demonstrated that plasma NFL levels are correlated with scores of UPDRS part III and MMSE in PD patients. Our results, with a relatively small number of patients, did not recapitulate these correlations. NFL levels in plasma centrifuged at RT were elevated in PD patients at the advanced stage, while PD patients at the early stage demonstrated higher NFL levels in plasma in refrigerated centrifugation. The reason for inconsistent results of NFL in advanced and early PD stages between RT and refrigerated centrifugation might be because of the small sample size tested, especially in the advanced stage (*n* = 18). A large prospective cohort or meta-analysis will be needed to consolidate the role of NFL in the disease progression of PD.

This study has some limitations. It should be noted that the sample size of this study was relatively small. The results should be further tested in well-powered cohorts. The levels of α-synuclein, which was produced by the IMR platform, should be validated by other quantitative technologies. The polymerization and phosphorylation of α-synuclein might not be properly identified. The parameters (α-synuclein and NFL), which had been shown to differentiate PD from atypical parkinsonism (Lee et al., 2006; Hansson et al., 2017; Lin et al., 2019), were not tested in patients with other parkinsonism or other neurological diseases. However, differentiation of PD from atypical parkinsonism was not the aim of our study. In the future, further studies should be conducted to examine if α-synuclein and NFL measured by applying the sample preparation used in our study can clearly distinguish PD from atypical parkinsonism. Nevertheless, this study evidently indicated that when processed in optimal conditions, K_2_-and K_3_-EDTA tubes used to collect blood for measurements of α-synuclein and NFL could display compatible results. Plasma should be prepared under RT centrifugation to lessen the reduction of α-synuclein. With standardized preanalytical sample preparations, both α-synuclein and NFL serve as potential biomarkers for PD. Our study results suggested that a consensus protocol of blood sample preparation was essential in quantifying α-synuclein levels if we would plan to use α-synuclein as a biomarker for PD.

## Data Availability Statement

The raw data supporting the conclusions of this article will be made available by the authors, without undue reservation.

## Ethics Statement

The studies involving human participants were reviewed and approved by the Institutional Review Boards of the Chang Gung Memorial Hospital. The patients/participants provided their written informed consent to participate in this study.

## Author Contributions

K-HC and C-MC contributed to conception and design of the study, organized the database, and finalized and approved the submitted version. K-CL and C-SL contributed reagents, materials, analysis tools. K-HC and S-YY performed the statistical analysis. K-HC wrote the first draft of the manuscript. All authors contributed to manuscript revision.

## Conflict of Interest

S-YY was employed by company MagQu Co., Ltd. The remaining authors declare that the research was conducted in the absence of any commercial or financial relationships that could be construed as a potential conflict of interest.

## Publisher’s Note

All claims expressed in this article are solely those of the authors and do not necessarily represent those of their affiliated organizations, or those of the publisher, the editors and the reviewers. Any product that may be evaluated in this article, or claim that may be made by its manufacturer, is not guaranteed or endorsed by the publisher.
