# Supplementary material for: Assessing Plasma Levels of α-Synuclein and Neurofilament Light Chain by Different Blood Preparation Methods
Source: Front Aging Neurosci. 2021 Nov 8;13:759182. doi: 10.3389/fnagi.2021.759182 (PMC8630588; doi:10.3389/fnagi.2021.759182)
Supplement: Supplementary file 1 [file Table_1.docx]

**Supplementary Table 1.** The limit of detection (LoD), analytical range, intra-assay (repeatability) and inter-assay (reproducibility) of the IMR assay to detect total α-synuclein and neurofilament light chain (NFL).

| Biomarker | Total α-synuclein | NFL | References |
| --- | --- | --- | --- |
| Repeatability  (Intra-assay) | Sample 1: 8.6%  Sample 2: 8.5% | Sample 1: 13.8%  Sample 2: 11.0% | CLSI EP-5-A3 |
| Reproducibility  (Inter-assay) | Sample 1: 2.67%  Sample 2: 0.53% | Sample 1: 1.89%  Sample 2: 1.31% | CLSI EP-5-A3 |
| LoD (pg/ml) | 0.0014 | 0.00018 | CLSI EP17-A2 |
| Analytical range  (pg/ml) | 0.0014 -1020 | 0.001-1000 | ICH Q2 (R1) |
| Specificity | No significant interference with:  Hemoglobin, bilirubin, intra lipid, uric acid, rheumatoid factor, albumin, acetylsalicylic acid, ascorbic acid, ampicillin sodium, quetiapine fumarate, galantamine hydrobromide, rivastigmine hydrogen tartrate, donepezil hydrochloride, or memantine hydrochloride | |  |
